# Understanding the behavioral determinants of adolescents’ water consumption: A cross-country comparative study

**DOI:** 10.1016/j.dialog.2023.100101

**Published:** 2023-01-18

**Authors:** Saskia C.M. Franken, Crystal R. Smit, Rebecca N.H. de Leeuw, Thabo J. van Woudenberg, William J. Burk, Kirsten E. Bevelander, Moniek Buijzen

**Affiliations:** a Faculty for Accounting, Finance, and Marketing, University of Aruba, J.E. Irausquinplein 4, Oranjestad, Aruba; b Erasmus School of Social and Behavioural Sciences, Erasmus University Rotterdam, P.O. Box 1738, 3000 DR Rotterdam, the Netherlands; c Behavioural Science Institute, Radboud University, P.O. Box 9104, 6500 HE Nijmegen, the Netherlands; d Radboud Institute for Health Sciences, Radboud University and Medical Centre, P.O. Box 9102, 6500 HC Nijmegen, the Netherlands

**Keywords:** Intrinsic motivation, Theory of planned behavior, Social norms, Sugar-sweetened beverages, Water consumption, Adolescents

## Abstract

Substituting the consumption of sugar-sweetened beverages (SSB) with that of water can have a positive effect on adolescents’ health. However, despite the attention on this topic in the Global North, it is relatively understudied in other regions of the world, such as the Caribbean. To guide the development of future interventions, understanding the factors determining water consumption among Caribbean adolescents is important. This study examined the behavioral determinants of water consumption among adolescents in Aruba (the Caribbean) and compared them to those in the Netherlands (Western Europe). We used a theoretical model that integrates the dominant theoretical perspectives in the field of public health, including theories of planned behavior, social norms, and intrinsic motivation. This cross-country study included 1,584 adolescents from Aruba and the Netherlands (52% girls; *M* = 12.34 years; *SD* = 2.14). The data were analyzed using regression analyses. This study found that in Aruba, adolescents with higher scores of intrinsic motivation, friends’ descriptive norms, attitudes, and behavioral control regarding water consumption drank more water. Moreover, the associations between water consumption and both intrinsic motivation as well as friends’ descriptive norms for adolescents in Aruba were stronger than those found in the Netherlands. These associations imply that it is even more important for Aruban adolescents than Dutch adolescents to be intrinsically motivated or to perceive their friends often consuming water to drink more water. The cross-country comparison implies that future interventions in Aruba aimed at increasing adolescents’ water consumption as an alternative to SSB should focus on enhancing their intrinsic motivation while considering their friends’ social norms.

## 1 Introduction

The consumption of sugar-sweetened beverages (SSBs) is associated with a greater risk of dental decay [1], weight gain [2], type 2 diabetes [3], and cardiovascular diseases [4]. Despite the evidence that high intake of SSB is related to negative health effects, the consumption of SSBs remains high among adolescents. In the Caribbean, the consumption of SSBs, including soda, sweetened juice drinks, sweetened milk drinks, and energy drinks is more than four times higher than in Western Europe [5]. Several approaches in health interventions can reduce SSB consumption [6]. One possible way is promoting water consumption, which has zero calories, as an alternative to SSB consumption. Several longitudinal studies have reported that replacing SSB with water exhibits a beneficial effect on children’s body weight [7,8]. However, the water intake of adolescents remains relatively low in numerous countries around the globe, especially in the Global South [[9], [10], [11]]. In Aruba, an island in the Caribbean, the water consumption among primary school children is also low [12].

While considerable research has been conducted on promoting water consumption and reducing the consumption of SSBs in the Global North [5,13], this topic remains under-researched in the Global South, especially in the Caribbean region. To improve health interventions in the Caribbean, it is crucial to gain a better understanding of the factors that determine Caribbean adolescents’ water consumption. Therefore, this study aims to understand the behavioral determinants of water consumption among adolescents in Aruba and to compare them with the Western European country of the Netherlands. This comparison provides guidance as to the design and implementation of future interventions in the Caribbean.

### 1.1 Comparing water consumption determinants across the Caribbean and Western Europe

Over the past decade, several European countries have examined the behavioral determinants of water consumption [14] and interventions aimed at promoting water consumption [13]. Particularly, researchers in the Netherlands have developed interventions to reduce SSB consumption by promoting water consumption [15,16]. Recently, an integrated theoretical model was applied by Smit et al. [17] to understand the determinants of water consumption among Dutch adolescents. However, to the best of our knowledge, researchers have not yet compared the determinants of water consumption in the Southern Caribbean and North-Western Europe. Furthermore, they have not examined the behavioral determinants of water consumption among Caribbean adolescents. This study aims to fill this knowledge gap by investigating whether the theoretical determinants applied in the model of Smit et al. [17] for the adolescents of the Netherlands can be generalized to adolescents of Aruba. To this end, we compare the determinants of water consumption between adolescents from Aruba and the Netherlands.

### 1.2 Theoretical determinants of water consumption

The integrated model applied by Smit et al. [17] incorporated determinants from various dominant theoretical perspectives in the field of public health. The model included determinants from the theory of planned behavior [18], social norms [19], and intrinsic motivation [20].

#### 1.2.1 Theory of planned behavior

The theory of planned behavior (TPB) [18] is one of the most commonly applied theoretical perspectives to predict behavioral changes in dietary patterns [21]. It states that behavior is predicted by an individual’s intention to perform a certain behavior, which is influenced by their (a) attitude toward the behavior, or the evaluation of the behavior, (b) subjective norms, or the perception of what others consider appropriate regarding the behavior, and (c) perceived behavioral control, or the perception that one is able to and is in control of their behavior. Several cross-sectional studies related to dietary patterns have demonstrated that these constructs directly affect behavior, and indirectly through behavioral intentions [21]. Thus, we will examine whether intention, attitude, subjective norms, and behavioral control are associated with adolescents’ water consumption in Aruba.

#### 1.2.2 Social norms

The integrated model described by Smit et al. [17] broadens the conceptualization of TPB’s subjective norms by distinguishing between different types and sources of normative influences. Recently, the literature on social norms has provided comprehensive insights into the social norm mechanisms influencing dietary intake [19]. This has highlighted the need to differentiate between the two types of social norms—descriptive norms (i.e., perceptions of the prevalence of others’ behavior) and injunctive norms (i.e., perceptions of whether others approve or disapprove of the behavior) [22]—as well as between the two main influencing sources: parents and friends [23]. The effects of normative influences vary across dietary behaviors [23,24]. Therefore, a broader conceptualization of the norms facilitates a deeper understanding of adolescents’ consumption behavior in Aruba. Thus, we integrated a broader conceptualization of the social norms construct with both descriptive and injunctive norms involving water consumption from both parents and friends.

#### 1.2.3 Intrinsic motivation

Smit et al. further expanded the TPB model [17] by including individuals’ motivation to engage in healthy behavior. The self-determination theory, a prominent theory of human behavior, shows that motivation, particularly intrinsic motivation, is an essential determinant of behavioral change [20]. Intrinsic motivation refers to the inner drive of individuals to perform a behavior because it is inherently interesting or enjoyable [20]. Moreover, individuals who are intrinsically motivated tend to adopt and maintain healthy lifestyle patterns over time [25]. Therefore, we examined intrinsic motivation as a potential determinant of water consumption among adolescents in Aruba.

### 1.3 Study aims

This study had two objectives. First, to identify the most important determinants for adolescents in Aruba, this study aimed to examine the determinants from various theoretical perspectives, namely intention, attitude, behavioral control, descriptive norms and injunctive norms of parents and friends, as well as intrinsic motivation. Second was to investigate whether the determinants of water consumption differ between adolescents in Aruba and those in the Netherlands.

## 2 Materials and methods

### 2.1 Aruba and the Netherlands

Aruba is separated from the Netherlands by the North Atlantic Ocean, yet, despite having relatively different cultural backgrounds, they share Dutch nationality. Aruba is an island situated in the Southern Caribbean and is the smallest of the four constituent countries of the Kingdom of the Netherlands. The official languages of Aruba are Papiamento and Dutch. Further, the Netherlands is a country located in North-Western Europe and is the largest of the four constituent countries of the Kingdom of the Netherlands. The official language of the Netherlands is Dutch. The Netherlands has a temperate maritime climate, while Aruba has a hot, semi-arid climate. Thus, the geographical location, land area size, primary language, and climate differ between the two countries; however, safe tap water is available in both countries.

### 2.2 Participants and procedure

The sample for this cross-country study consisted of three similar datasets: two datasets from Aruba and one from the Netherlands. The first dataset from Aruba was collected from six secondary schools [*n* = 398; see Franken et al. [26] for methodology description]. The second Aruban dataset consisted of the baseline data from a water promotion intervention study conducted in six primary schools [*n* = 394; see Franken et al. [12] for project description]. The dataset from the Netherlands was collected from 13 primary schools (*n* = 355) and eight secondary schools (*n* = 437) that participated in the first wave of the *MyMovez* project [see Bevelander et al. [27] for the study protocol]. Consequently, 1,584 adolescents were included in the analysis, 52% of whom were female. Moreover, the adolescents’ ages ranged between 8 and 18 years (*M* =12.34; *SD* = 2.14).

Before each of the three datasets was collected, school directors were asked to provide their consent to conduct the study at their schools. In both countries, informed consent was obtained from parents and assent from adolescents [see Bevelander et al. [27], Franken et al. [12,26] for the protocol and detailed procedures]. In Aruba, the majority of questionnaires were completed in Papiamento. In the Netherlands, questionnaires were completed in Dutch. The Ethics Committee of the Faculty of Social Sciences at Radboud University approved the data collection procedures in Aruba (ECSW2014-1003-203) and the ethical review board of the European Research Council (617253) approved the *MyMovez* project in the Netherlands.

### 2.3 Measures

An overview of all the study variables (water consumption, intention, attitude, behavioral control, descriptive and injunctive norms of parents, descriptive and injunctive norms of friends, as well as intrinsic motivation) and the covariates (country of residence, thirst level, sex, age, and SSB consumption) are presented in Table 1. Since the constructs used across the three datasets are mostly similar, the three datasets were merged after adjustments.

**Table 1:** Overview of measures.

Measure	Primary schools, Aruba	Secondary schools, Aruba	Primary and secondary schools, the Netherlands
Water consumption: a	How much water do you drink on (1) a normal school day and (2) a normal weekend day? The questionnaire illustrated that a glass also represents a bottle, a can, or a package to facilitate participants’ quantity estimation. Answer categories 0 (*zero glasses*) to 8 (*eight glasses or more*). b	Idem.	How much water did you drink yesterday? This question was asked on three different days. The questionnaire illustrated that a glass also represents a bottle, a can, or a package to facilitate participants’ quantity estimation. Answer categories 0 (*zero glasses*) to 7 (*seven glasses or more*). b
Behavioral intention: c	Do you intend to drink more water? 1 (*no, certainly do not*) to 4 (*yes, certainly do*).	Idem.	Question idem, 1 (*no, certainly do not*) to 6 (*yes, certainly do*).
Attitude:	I find drinking water… (a) 1 (*very unpleasant*) to 4 = (*very pleasant*), and (b) 1 (*very distasteful*) to 4 (*very tasteful*). b Spearman-Brown = .74	Idem.	Idem. Spearman-Brown = .85
Behavioral control: c	Do you think you will succeed in drinking more water? 1 (*no, certainly do not*) to 4 (*yes, certainly do*).	Question idem, 1 (*not certain*) to 4 (*very certain*).	Question idem, 1 (*no, certainly do not*) to 6 (*yes, certainly do*).
Descriptive norm parents: c	How often does your (1) father, (2) mother drink water? 1 (*never*) to 4 (*always*). d Spearman-Brown = .51	Does your (1) father, (2) mother ever drink water? 1 (*no, never*) to 4 (*yes, often*). d	How often do your parents drink water? 1 (*never*) to 6 (*always*).
Descriptive norm friends: c	How often do your friends drink water? 1 (*never*) to 4 (*always*).	How many of your friends drink water? 1 (*nobody*) to 4 (*most or all of them*).	How often do your friends drink water? 1 (*never*) to 6 (*always*).
Injunctive norm parents: c	How often does your (1) father, (2) mother approve that you drink water? 1 (*never*) to 4 (*always*). d Spearman-Brown = .85	Does your (1) father, (2) mother approve that you drink water? 1 (*no, absolutely not*) to 4 (*yes, a lot*). d	Do you experience that your parents think you should drink water? 1 (*no, certainly do not*) to 6 (*yes, certainly do*).
Injunctive norm friends: c	How often do your friends approve that you drink water? 1 (*never*) to 4 (*always*).	Do your friends approve that you drink water? 1 (*no, absolutely not*) to 4 (*yes, a lot*).	Do you experience that your friends think you should drink water? 1 (*no, certainly do not*) to 6 (*yes, certainly do*)
Intrinsic motivation: c	How often do you drink water because you… (a) like it?, (b) enjoy it?, (c) think it is pleasant?, (d) choose to do so yourself? 1 (*never*) to 4 (*always*). b Spearman-Brown = .86	Do you drink water because you… a, b, c idem. Question d: always do so? 1 (*not true*) to 4 (*very true*). b	Do you drink water because you… a, b, c idem. Question d: want this yourself? 1 (*no, certainly do not*) to 6 (*yes, certainly do*). b Spearman-Brown = .85
Thirst level:	How thirsty are you at this moment? Thirst level was measured with a Visual Analog Scale (VAS): 0 cm (*I am not thirsty*) to 15 cm (*I am very thirsty*).	Question idem, 1 (*not thirsty*) to 4 (*very thirsty*). e	Question idem, VAS: 0 cm (*not thirsty*) to 15 cm (*very thirsty*).
Sex:	Coded 0 (*boys*), 1 (*girls*).	Idem.	Idem.
Age:	Open question.	Idem.	Idem.
Sugar-sweetened beverage consumption: a	How many glasses of (a) juice drinks, (b) soda, and (c) energy and sports drinks do you drink on (1) a normal school day and (2) a normal weekend day? The same illustration used for water consumption was applied here. Answer categories 0 (*zero glasses*) to 8 (*eight glasses or more*). b	Idem.	How many glasses of (a) juice drinks, (b) soda, and (c) energy, and (d) sports drinks do you drink? This question was asked on three different days. The same illustration used for water consumption was applied here. Answer categories 0 (*zero glasses*) to 7 = (*seven glasses or more*). b

a Before aggregating the Aruban and Dutch databases, Aruban participants who answered 8 were recoded with 7 such that the final sample consisted of an equal score range.

b A total score was attained by averaging the scores for the subitems.

c Before merging the databases, the scores for the Aruban adolescents were divided by 4 and multiplied by 6 to attain an equal score range.

d Before aggregating the databases, a parent score was constructed for the Aruban adolescents by averaging the father and mother items.

e Before aggregating the databases, the 4-point scale answers were divided by 4 and multiplied by 15 to acquire an equal range of answer scores in the final sample.

### 2.4 Statistical analysis

The study variables were analyzed using descriptive statistics. Pearson correlations were performed for Aruba and the Netherlands to examine bivariate associations between all study variables. The primary analyses consisted of two multiple regression analyses, in which four covariates (sex, age, thirst, and SSB consumption) and the focal variables (intention, attitude, behavioral control, descriptive and injunctive norms of parents and friends, and intrinsic motivation) were included. First, the Aruban samples (*n* = 792) were analyzed to determine the main effects of each behavioral determinant of adolescents’ water consumption. The second analysis was performed on all three samples (*N* = 1,584) and included the same variables as the first analysis, as well as the main effects of country, and X interactions between country and the behavioral determinants. Furthermore, interaction terms were created between the country of residence and the mean-centered behavioral determinants. Statistically significant interactions were further examined using simple slope analyses across two levels of a moderator (low level -1 *SD*; high level +1 *SD*). All analyses were performed in SPSS version 28 (SPSS, Inc., Chicago, IL, U.S.), and the SPSS PROCESS macro [28] (version 4) was used for probing the statistically significant interactions.

## 3 Results

### 3.1 Descriptive statistics

Table 2 presents the mean and standard deviation of all the study variables.^1^ The *t*-tests showed that all variables differed significantly between the two countries (*p* < .05, two-tailed), except for thirst (*p* = .44). Moreover, Pearson’s correlations in Table 3 indicate significant positive associations between Aruban adolescents’ water consumption and their behavioral determinants—intention, attitude, behavioral control, parents’ and friends’ descriptive norms, parents’ injunctive norms, and intrinsic motivation. Table 4 shows Pearson’s correlations for Dutch adolescents, indicating significant positive associations between water consumption and their behavioral determinants—attitude, behavioral control, parents’ and friends’ descriptive norms, and intrinsic motivation.

**Table 2:** Descriptive statistics for the total sample and by country of residence.

	Total		Aruba		the Netherlands	
	*N* = 1,584		*n* = 792		*n* = 792	
Variable	*M*^b^	*SD*	*M*	*SD*	*M*	*SD*
Thirst level	6.47 (0–15)	3.57	6.54 (0–15)	3.74	6.40 (0–15)	3.38
SSB consumption a (i.e., glasses)	1.11 (0–7)	1.14	1.80 (0–7)	1.14	0.41 (0–7)	0.58
Water consumption (i.e., glasses)	3.76 (0–7)	1.83	4.49 (0–7)	1.75	3.02 (0–7)	1.59
Behavioral intention	4.59 (1–6)	1.43	4.99 (2–6)	1.13	4.10 (1–6)	1.60
Attitude	3.33 (1–4)	0.69	3.37 (1–4)	0.64	3.29 (1–4)	0.73
Behavioral control	4.94 (1–6)	1.27	4.87 (2–6)	1.21	5.03 (1–6)	1.33
Descriptive norm parents	4.82 (1–6)	1.14	5.21 (2–6)	0.90	4.33 (1–6)	1.22
Descriptive norm friends	4.01 (1–6)	1.29	4.32 (2–6)	1.29	3.63 (1–6)	1.19
Injunctive norm parents	4.98 (1–6)	1.36	5.16 (2–6)	1.17	4.74 (1–6)	1.55
Injunctive norm friends	3.61 (1–6)	1.67	3.92 (2–6)	1.53	3.20 (1–6)	1.76
Intrinsic motivation	4.52 (1–6)	1.27	4.40 (2–6)	1.24	4.64 (1–6)	1.29

a Sugar-sweetened beverages.

b Ranges are in parentheses.

**Table 3:** Pearson correlations among the variables for adolescents in Aruba.

Variable	1	2	3	4	5	6	7	8	9	10	11	12
1. Thirst level	..	.17⁎⁎	.11⁎⁎	.11⁎⁎	.09⁎	.04	-.01	.09⁎	.02	-.01	.00	.01
2. Age			.10⁎⁎	.07⁎	-.01	-.16⁎⁎	-.25⁎⁎	.39⁎⁎	.19⁎⁎	-.16⁎⁎	.07	-.21⁎⁎
3. SSB consumption^a^				.14⁎⁎	-.07	-.16⁎⁎	-.10⁎⁎	.06	.01	-.03	.01	-.15⁎⁎
4. Water consumption					.12⁎⁎	.32⁎⁎	.21⁎⁎	.17⁎⁎	.22⁎⁎	.10⁎⁎	.00	.38⁎⁎
5. Behavioral intention						.27⁎⁎	.43⁎⁎	.12⁎⁎	.00	.19⁎⁎	.02	.32⁎⁎
6. Attitude							.39⁎⁎	.05	.10⁎⁎	.21⁎⁎	.00	.63⁎⁎
7. Behavioral control								-.01	.01	.25⁎⁎	-.00	.44⁎⁎
8. Descriptive norm parents									.34⁎⁎	.11⁎⁎	.07⁎	.13⁎⁎
9. Descriptive norm friends										.03	.10⁎⁎	.16⁎⁎
10. Injunctive norm parents											.02	.33⁎⁎
11. Injunctive norm friends												.04
12. Intrinsic motivation												..

*Note*. *N* = 792.

a Sugar-sweetened beverages.

⁎ *p* < .05.

⁎⁎ *p* < .01.

**Table 4:** Pearson correlations among the variables for adolescents in the Netherlands.

Variable	1	2	3	4	5	6	7	8	9	10	11	12
1. Thirst level	..	-.10⁎⁎	.12⁎⁎	.03	.03	-.02	.06	-.06	-.04	-.02	.02	-.00
2. Age			-.01	.16⁎⁎	-.12⁎⁎	-.05	-.05	-.07	.00	-.06	.01	-.01
3. SSB consumption^a^				.09⁎⁎	.09⁎	-.17⁎⁎	-.06	-.07	.05	-.12⁎⁎	.13⁎⁎	-.09⁎
4. Water consumption					.01	.24⁎⁎	.13⁎⁎	.12⁎⁎	.09⁎	.07	.08	.21⁎⁎
5. Behavioral intention						.25⁎⁎	.42⁎⁎	.21⁎⁎	.20⁎⁎	.23⁎⁎	.25⁎⁎	.29⁎⁎
6. Attitude							.34⁎⁎	.18⁎⁎	.19⁎⁎	.14⁎⁎	.13⁎⁎	.67⁎⁎
7. Behavioral control								.21⁎⁎	.18⁎⁎	.19⁎⁎	.18⁎⁎	.36⁎⁎
8. Descriptive norm parents									.47⁎⁎	.35⁎⁎	.18⁎⁎	.21⁎⁎
9. Descriptive norm friends										.18⁎⁎	.29⁎⁎	.26⁎⁎
10. Injunctive norm parents											.49⁎⁎	.09⁎
11. Injunctive norm friends												.16⁎⁎
12. Intrinsic motivation												..

*Note*. *N* = 792.

a Sugar-sweetened beverages.

⁎ *p* < .05.

⁎⁎ *p* < .01.

### 3.2 Main analyses

#### 3.2.1 Water consumption determinants for Aruban adolescents

Table 5 presents the results of the first regression analysis. This regression investigated the theoretical determinants of adolescents’ water consumption in Aruba. The model showed that the variables explained 25% of the variance in water consumption for adolescents in Aruba (*F*[12, 726] = 20.11, *p* = <.001). Furthermore, the analysis revealed significant associations between the water consumption of adolescents in Aruba and their intrinsic motivation, attitude, descriptive friends’ norms, and behavioral control. Accordingly, adolescents with higher scores for intrinsic motivation, attitude, friends’ norms, and behavioral control consumed more water.

**Table 5:** Regression results between behavioral determinants and water consumption of Aruban adolescents.

Determinant	*b*	*SE B*	*β*	*p*	95% CI b
Constant	-1.35	0.61	..	.026	-2.55, -0.16
Thirst level	0.03	0.02	.06	.080	-0.00, 0.06
Sex a	-0.29	0.11	-.08	.012	-0.51, -0.06
Age	0.09	0.03	.12	.001	0.04, 0.15
Sugar-sweetened beverage consumption	0.27	0.05	.18	<.001	0.17, 0.37
Behavioral intention	-0.05	0.06	-.03	.376	-0.16, 0.06
Attitude	0.30	0.12	.11	**.009**	0.08, 0.53
Behavioral control	0.12	0.06	.09	**.029**	0.01, 0.23
Descriptive norm parents	0.05	0.07	.03	.501	-0.10, 0.20
Descriptive norm friends	0.16	0.05	.12	**<.001**	0.06, 0.25
Injunctive norm parents	-0.01	0.05	-.01	.813	-0.12, 0.09
Injunctive norm friends	-0.03	0.04	-.03	.401	-0.10, 0.04
Intrinsic motivation	0.45	0.06	.32	**<.001**	0.32, 0.57

*Note. N* = 792.

Significant variables (*p* < .05) appear in bold.

*R* = .50, *R*^*2*^ = .25, *F*(12, 726) = 20.11, *p* = <.001.

a 0 = boys, 1 = girls.

b Confidence interval.

#### 3.2.2 Comparing water consumption determinants between Aruba and the Netherlands

The results of the second regression analysis are presented in Table 6. This analysis examined the differences between water consumption determinants among adolescents in Aruba (*n* = 792) and the Netherlands (*n* = 792). The overall model was statistically significant (*R* = .60, *R*^*2*^ = .36, *F*(21, 1243) = 33.92, *p* = < .001). Water consumption in both Aruba and the Netherlands was significantly positively associated with attitude (*p* = .006), behavioral control (*p* = .019), intrinsic motivation (*p* = < .001), and descriptive norm of friends (*p* = < .001). Moreover, the interaction term between intrinsic motivation and the country was significant for water consumption (*b* = -0.34, *SE* = 0.10, *ß* = -.15, *p* = < .001, 95% CI [-0.53 to -0.15]). In addition, the interaction term between friends’ descriptive norm and the country was significant for water consumption (*b* = -0.17, *SE* = 0.08, *ß* = -.08, *p* = .030, 95% CI [-0.33 to -0.02]).

**Table 6:** Regression analysis examining the interaction effects between country of residence and behavioral determinants on water consumption.

Determinant	*b*	*SE B*	*β*	*p*	95% CI c
Constant	-1.63	0.57	..	.005	-2.75, -0.50
Thirst level	0.02	0.01	.04	.102	-0.00, 0.04
Sex a	-0.22	0.09	-.06	.009	-0.39, -0.05
Age	0.12	0.03	.15	<.001	0.07, 0.17
Sugar-sweetened beverage consumption	0.27	0.05	.17	<.001	0.18, 0.36
Country of residence b	-0.97	0.13	-.26	<.001	-1.22, -0.72
Behavioral intention	-0.06	0.06	-.04	.317	-0.16, 0.05
Attitude	0.31	0.11	.11	.006	0.09, 0.53
Behavioral control	0.13	0.06	.09	.019	0.02, 0.24
Descriptive norm parents	0.03	0.07	.02	.682	-0.11, 0.17
Descriptive norm friends	0.15	0.05	.11	<.001	0.06, 0.24
Injunctive norm parents	-0.01	0.05	-.01	.857	-0.11, 0.09
Injunctive norm friends	-0.03	0.04	-.03	.348	-0.10, 0.04
Intrinsic motivation	0.46	0.06	.31	<.001	0.33, 0.58
Country X Behavioral intention	-0.05	0.07	-.03	.505	-0.19, 0.09
Country X Attitude	0.16	0.17	.04	.344	-0.17, 0.49
Country X Behavioral control	-0.09	0.08	-.04	.285	-0.24, 0.07
Country X Descriptive norm parents	0.13	0.10	.06	.167	-0.06, 0.32
Country X Descriptive norm friends	-0.17	0.08	-.08	**.030**	-0.33, -0.02
Country X Injunctive norm parents	0.03	0.07	.02	.643	-0.11, 0.18
Country X Injunctive norm friends	0.05	0.06	.03	.428	-0.07, 0.16
Country X Intrinsic motivation	-0.34	0.10	-.15	**<.001**	-0.53, -0.15

*Note. N* = 1,584.

Significant variables (*p* < .05) appear in bold.

*R* = .60, *R*^*2*^ = .36, *F*(21, 1243) = 33.92, *p* = < .001.

a 0 = boys, 1 = girls.

b 0 = Aruba, 1 = the Netherlands.

c Confidence interval.

**Fig. 1:**
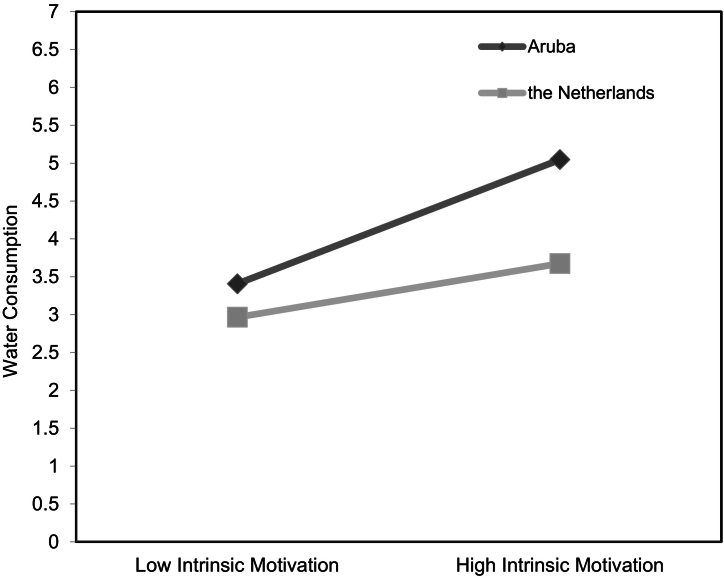
Associations between intrinsic motivation and water consumption for adolescents residing in Aruba and the Netherlands.

We performed simple slopes analyses to further interpret these two statistically significant interactions. In Fig. 1, the significant interaction is depicted with water consumption (controlled for thirst level, sex, age, and SSB consumption) on the *y*-axis, low (-1 *SD*) and high (+1 *SD*) intrinsic motivation on the *x*-axis, as well as separate regression slopes for adolescents from each country. Both simple slopes revealed a significant positive association between intrinsic motivation and water consumption, with intrinsic motivation being more strongly associated with water consumption for adolescents in Aruba (*b* = 0.64, *SE* = 0.05, *t* = 14.13, *p =* < .001, 95% CI [0.55, 0.73]) than for adolescents in the Netherlands (*b* = .28, *SE* = 0.04, *t* = 6.53, *p =* < .001, 95% CI [0.19, 0.36]).

**Fig. 2:**
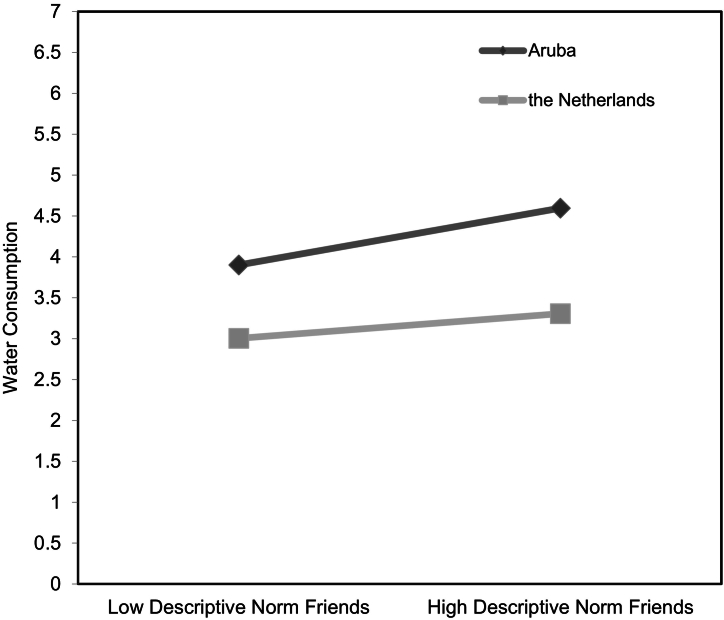
Associations between descriptive norm of friends and water consumption for adolescents residing in Aruba and the Netherlands.

Fig. 2 presents the significant interaction between water consumption (controlled for thirst level, sex, age, and SSB consumption) on the *y*-axis, low (-1 *SD*) and high (+1 *SD*) descriptive friends’ norms on the *x*-axis, and two regression slopes representing adolescents from each country. The simple slopes revealed significant positive associations between descriptive friends’ norms and water consumption, with descriptive norms being more strongly related to water consumption among Aruban adolescents (*b* = 0.27, *SE* = 0.05, *t* = 5.82, *p =* < .001, 95% CI [0.18, 0.36]) compared with Dutch adolescents (*b* = 0.12, *SE* = 0.05, *t* = 2.19, *p =* .029, 95% CI [0.01, 0.22]).

## 4 Discussion

The present study was the first cross-country study to examine the determinants of water consumption among adolescents on an under-researched Caribbean island (Aruba) and to compare them with those of a well-researched Western European country (the Netherlands). Several theoretical constructs were identified as unique behavioral determinants of water consumption among adolescents in Aruba. Our findings showed that intrinsic motivation to consume water was the most important determinant for Aruban adolescents’ water consumption. In addition, we found that Aruban adolescents consumed more water when they had a favorable attitude, had a higher perception that they were able to consume more water, and had a higher perception that their friends often consumed water. Furthermore, when comparing the determinants between Aruba and the Netherlands, the associations between water consumption and both intrinsic motivation and friends’ descriptive norms were stronger for adolescents in Aruba than those in the Netherlands.

### 4.1 The most important determinants of water consumption among Aruban adolescents

Water consumption among Aruban adolescents is uniquely explained by higher levels of intrinsic motivation, a positive attitude towards water consumption, higher levels of behavioral control, and higher descriptive norms of friends’ water consumption. Among these determinants, intrinsic motivation to drink water was the most important. This finding contributes to the accumulating evidence that intrinsic motivation plays an important role in explaining health-related behaviors, including those of Aruban adolescents [20,29]. Additionally, this is consistent with the findings in the Netherlands, indicating that intrinsically motivated adolescents consumed more water over time [17].

Furthermore, friends’ descriptive norms were associated with the water consumption of Aruban adolescents, rather than parents’ descriptive norms or the injunctive norms of friends or parents. This provides further evidence that it is important to differentiate between the types (descriptive, injunctive) and sources (parents, friends) of norms for understanding adolescents’ water consumption behavior [17,22,23]. The finding that adolescents consume more water when they perceive that their friends do is consistent with research showing that adolescents tend to follow the modeled consumption behavior of their friends [24]. However, it is possible that only friends are a social source of influence because adolescents in this age group spend more time at school and are therefore more exposed to their friends’ behavior than that of their parents [30].

In addition, behavioral attitude and control were found to influence the water consumption of Aruban adolescents. These direct associations between the constructs of the theory of planned behavior and the reported behavior of Aruban adolescents were similar to those found in previous literature [21]. These findings indicate that Aruban adolescents tend to consume more water when they have a more favorable attitude and perceive that they are capable of drinking more water.

Moreover, there was no association between adolescents’ intentions to consume water and their actual consumption. This finding supports the “intention-behavior gap” that has been found in other studies [31], particularly with regard to water consumption [17]. This gap implies that individuals often intend to pursue a healthy behavior, but do not act upon their intentions [31]. This study shows that having the intention to consume more water does not imply that individuals actually consume more water.

### 4.2 Differences between determinants of Aruba and the Netherlands

The associations of intrinsic motivation and descriptive norms of friends with water consumption were stronger for adolescents from Aruba compared to those from the Netherlands. This difference in strength indicates that it is even more important for Aruban adolescents to be intrinsically motivated or to perceive their friends often consuming water to be inclined to drink even more water than Dutch adolescents. An explanation for a stronger association between intrinsic motivation and water consumption could be that the minimum physiological needs for water may vary according to geographical location and climate [11]. Consequently, the stronger association of motivation could be due to Aruba’s hot, semi-arid climate that incites an innate physiological need to quench thirst with water compared to the more temperate climate of the Netherlands [12].

In terms of descriptive norms, despite the fact that these countries are constituent members of the Kingdom of the Netherlands, there may be relative culture-based value differences. Relatively speaking, Arubans value being connected to other individuals within their social environment, compared to Dutch people, who have more individualistic values and weaker social ties [32]. Consequently, these differences in values explain why adolescents in the collectivistic Aruban community are more inclined to consume water when they observe their friends doing so than the adolescents from the individualistically inclined Dutch community [33]. Thus, the results suggest an amplified interplay between environmental factors and adolescents’ behavioral determinants that influence water consumption. In the future, this study could be replicated, and the relationship between water consumption and these climactic and culture-based value factors could be examined.

Although a further examination of water and SSB consumption is beyond the scope of our research aims, and caution should be taken with the interpretation of the self-reported units (i.e., glasses) of these fluids, the results reveal that consumption patterns differ between Aruba and the Netherlands. This study reports (Table 2) that despite Aruban adolescents reporting consuming more water (4.49 units, i.e., glasses, during a normal school day) compared to Dutch adolescents (3.02 units), the amounts of water consumed in both countries are nevertheless insufficient. Regarding fluid intake, Aruban health authorities recommend only consuming water or beverages containing no calories [34]. They recommend that children 9 to 12 years and adolescents 13 to 18 consume 6 to 8 and 7 to 8 glasses of fluids containing no calories respectively. Thus, this study also highlights that adolescents do not meet the recommended guidelines for beverage consumption. Inadequate water consumption has also been detected in earlier Aruban studies [12,35] and in many other countries around the globe [11], which further underscores the need to promote this behavior in the future.

Moreover, our SSB consumption findings for Aruba and the Netherlands align with global and regional consumption patterns. Our findings reveal that the SSB consumption pattern for Aruban adolescents is more than four times higher than in the Netherlands, respectively 1.8 and 0.41 units (i.e., glasses) of SSBs during a normal school day. This finding is similar to the global patterns laid out by Singh et al. [5], demonstrating that SSB consumption in the Caribbean is more than four times as much as in Western European countries—1.93 and 0.39 servings of SSBs, respectively. Especially for Aruba, this is worrisome because a can of SSB contains around 10 teaspoons of free sugar, while the World Health Organization encourages countries to reduce free sugar intake among children and adults to less than six teaspoons to prevent health-related complications [36,37]. These high-SSB consumption patterns in the Caribbean underline the recognized urgency among policymakers to facilitate healthier lifestyles in the Caribbean [[38], [39], [40], [41]].

### 4.3 Strengths and limitations

This study addressed a gap in the current literature by providing theory-based knowledge about the determinants of water consumption for the Caribbean island of Aruba. In doing so, this study reveals that the theoretical determinants applied in the model of Smit et al. [17] for the adolescents of the Netherlands can be generalized to adolescents of Aruba. Therefore, this study may provide theory-based support for policymakers, institutions, and researchers in future public health promotion activities worldwide, especially in the Caribbean region.

Several methodological limitations should be considered. The cross-sectional design prevents us from establishing causal relationships between the theory-based determinants and water consumption. Therefore, a future longitudinal study may provide insight into the directionality among the variables.

Another limitation of this study is the generalizability of its findings. Although a quarter of all Aruban primary and secondary schools participated in the study, caution should be exercised when generalizing our findings. Additionally, it may not be possible to generalize the water consumption determinants that play a role in Aruba to other islands in the Caribbean because of factors such as distinctive sociodemographic backgrounds, socioeconomic status, ethnic backgrounds, and educational levels [14]. Consequently, generalizing this study’s findings for the population of adolescents in Aruba or the Caribbean may have limitations.

Furthermore, although safe tap water is available in Aruba and the Netherlands, this is not always the case in other countries. In some Caribbean islands and other countries in the Global South, access to—and affordability and availability of—safe drinking water are limited [[9], [10], [11]]. Thus, this issue potentially limits our findings’ generalizability. Therefore, future research on ensuring safe drinking water for all is required before the implementation of water consumption promotion campaigns for positive health outcomes in the Global South can be considered [[9], [10], [11]].

### 4.4 Future research implications

This study established a focused groundwork for future health-related research in the Caribbean region by building on the knowledge of water consumption from the Global North. The cross-country findings indicate that promoting water consumption appears to be a promising research route for Aruba to encourage healthier lifestyles. Accordingly, Aruba should implement interventions that integrate motivational methods derived from the self-determination theory [20,42] and social norm principles [19,23,24].

The “Share H2O” program in the Netherlands is a social network-based behavioral-change intervention approach that incorporates motivational techniques and social norm mechanisms [16]. This program exposed participants to peer influencers in their social network who promoted water consumption. The peer influencers’ intrinsic motivation to drink more water and model this behavior to others was encouraged during the intervention training sessions. In the future, tailoring this program could lead to greater efficacy in Aruba than in the Netherlands, considering the differences between Aruba and the Netherlands in terms of the determinants of water consumption. Furthermore, there are other types of interventions and complementary measures that reduce SSB consumption or increase water consumption effectively [6], which can be considered in future research.

### 4.5 Conclusion

This study highlights that intrinsic motivation to drink water is the most important determinant of water consumption among Aruban adolescents. In addition, friends’ descriptive norms, attitudes, and behavioral control are considered to be important for Aruba. It has been found that Aruban adolescents with higher levels of these determinants consume more water. Furthermore, the comparison between Aruba and the Netherlands in terms of the determinants has demonstrated that there is a stronger association between intrinsic motivation and friends’ descriptive norms on the one hand and water consumption on the other hand for Aruba than for the Netherlands. According to our findings, water consumption promotion interventions that focus on increasing the intrinsic motivation to drink water and the social norms concerning water drinking are likely to be effective in Aruba. Finally, the findings of this study highlight the importance of extending the research area to include more countries from the Global South. By moving beyond countries from the Global North, we can gain knowledge that can contribute to equality and a healthy environment for all human beings.

## Role of the funding source

The University of Aruba, the employer of the first author, SCMF, financed the research, leading to these results. This research did not receive any specific grant from funding agencies in the public, commercial, or not-for-profit sectors. Authors CRS, RNHL, TJW, WJB, KEB, and MB are affiliated with the “*MyMovez*: Social Network Implementation of Health Campaigns,” financed by the European Research Council under the European Union’s Seventh Framework Programme (FP7/2007–2013)/ERC Grant Agreement 617253.

## Research data sharing

The deidentified participant dataset analyzed for the present study is available from the corresponding author upon reasonable request. Data of the *MyMovez* project are stored in the repository of DANS [43].

## Authors' contributions

SCMF is the primary researcher on the study and the corresponding author. SCMF, CRS, and MB conceptualized and designed the study. SCMF coordinated school visits and collected data in Aruba with the assistance of other data collectors. CRS, KEB, and TJW collected data in the Netherlands. SCMF analyzed the collected data, WJB provided statistical advice, and CRS directly accessed and verified the data and analyses, which were reviewed by MB and RNHL. SCMF wrote the manuscript and CRS, RHNL, KEB and MB critically reviewed it. All the authors reviewed the content and accept responsibility for the submitted version.

## Consent to participate and ethics approval

In Aruba and the Netherlands, informed consent was collected from parents and assent from adolescents. The Ethics Committee of the Faculty of Social Sciences at Radboud University approved the data collection procedures in Aruba (ECSW2014-1003-203) and the ethical review board of the European Research Council (617253) approved the *MyMovez* project from the Netherlands.

## Declaration of competing interest

The authors declare that they have no known competing financial interests or personal relationships that could have appeared to influence the work reported in this paper.

## Footnotes

1 The skewness of the covariate SSB consumption was 1.58, indicating that the distribution was positively skewed. However, our study’s results remained the same irrespective of whether untransformed or transformed scores were used. Therefore, the untransformed measure is used in all the analyses.
